# Caspase-8 deficiency induces a switch from TLR3 induced apoptosis to lysosomal cell death in neuroblastoma

**DOI:** 10.1038/s41598-021-89793-1

**Published:** 2021-05-19

**Authors:** Marie-Anaïs Locquet, Gabriel Ichim, Joseph Bisaccia, Aurelie Dutour, Serge Lebecque, Marie Castets, Kathrin Weber

**Affiliations:** 1 Childhood Cancers and Cell Death Laboratory, Cancer Research Center of Lyon (CRCL), INSERM 1052, CNRS 5286, Lyon, France; 2 Cancer Cell Death Laboratory, Part of LabEx DEVweCAN, Cancer Initiation and Tumoral Cell Identity Department, CRCL, Lyon, France; 3 Service D’Anatomie Pathologique, Hospices Civils de Lyon, Hôpital Lyon Sud, Pierre-Bénite, France

**Keywords:** Apoptosis, Targeted therapies, Paediatric cancer

## Abstract

In cancer cells only, TLR3 acquires death receptor properties by efficiently triggering the extrinsic pathway of apoptosis with Caspase-8 as apical protease. Here, we demonstrate that in the absence of Caspase-8, activation of TLR3 can trigger a form of programmed cell death, which is distinct from classical apoptosis. When TLR3 was activated in the Caspase-8 negative neuroblastoma cell line SH-SY5Y, cell death was accompanied by lysosomal permeabilization. Despite caspases being activated, lysosomal permeabilization as well as cell death were not affected by blocking caspase-activity, positioning lysosomal membrane permeabilization (LMP) upstream of caspase activation. Taken together, our data suggest that LMP with its deadly consequences represents a “default” death mechanism in cancer cells, when Caspase-8 is absent and apoptosis cannot be induced.

## Introduction

Lysosomal membrane permeabilization or LMP is the critical and initiating event that induces lysosomal cell death (LCD), though the onset and subsequent mechanisms remain unclear^1^. As lysosomes represent the degradative endpoint of autophagy and endocytotic pathways they contain various types of proteolytic enzymes, like cathepsins, which facilitate not only recycling of cellular components and the breakdown of major macromolecules, but also the degradation of superfluous or damaged organelles such as mitochondria^2^. LMP causes the release of lysosomal proteases, like for example cathepsins into the cytosol, where they can cleave several cellular targets^3^. For example, in the cytosol, cathepsins can cleave BID to truncated BID, which leads to the permeabilization of the mitochondrial membrane with subsequent cytochrome *c* release and activation of caspases^4^.

Lysosomes are also an important signaling hub for innate immunity, which is integrated by several Toll-like receptors like for example TLR3. Sensing viral dsRNA within the lysosomal lumen, TLR3 mounts an innate immune response by activating NFκB and IRF3^5^. In cancer cells only, TLR3 acquires additional death receptor properties, which has been reported for an increasing range of different cancers, such as neuroblastoma (NB), hepatocarcinoma, lung cancer or mesothelioma^6–9^. Thus, TLR3 acts not only as an adjuvant in cancer therapy due to its innate inflammatory signaling capacity^10^, but also has the potential of a novel therapeutic target to selectively kill a broad range of cancer cells.

Mechanistically TLR3 activates the extrinsic apoptotic pathway, with Caspase-8 as an initiator caspase^11,12^. Caspase-8 subsequently activates the caspase cascade directly by cleaving effector caspases like Caspase-3, and/or indirectly by amplifying death signaling by engaging the intrinsic apoptotic pathway through Caspase-8-dependent cleavage of BID to tBID. Besides apoptosis, TLR3 can also induce a different, caspase-independent form of programmed cell death (PCD), called necroptosis. During necroptosis, RIPK3 assembles into a signaling complex, which triggers the conversion of dormant MLKL into a plasma-membrane permeabilizing pore^13^.

Here, we describe for the first time that activation of TLR3 induces an alternative form of (PCD), characterized by the permeabilization of lysosomes as the initiating cell death event. This programmed cell death mode occurs in the absence of Caspase-8/apoptosis and RIPK3/necroptosis and can represents a “default” death mechanism.

## Results

### TLR3 can induce cell death in Caspase-8′ deficient SH-SY5Y neuroblastoma cells

**Figure 1:**
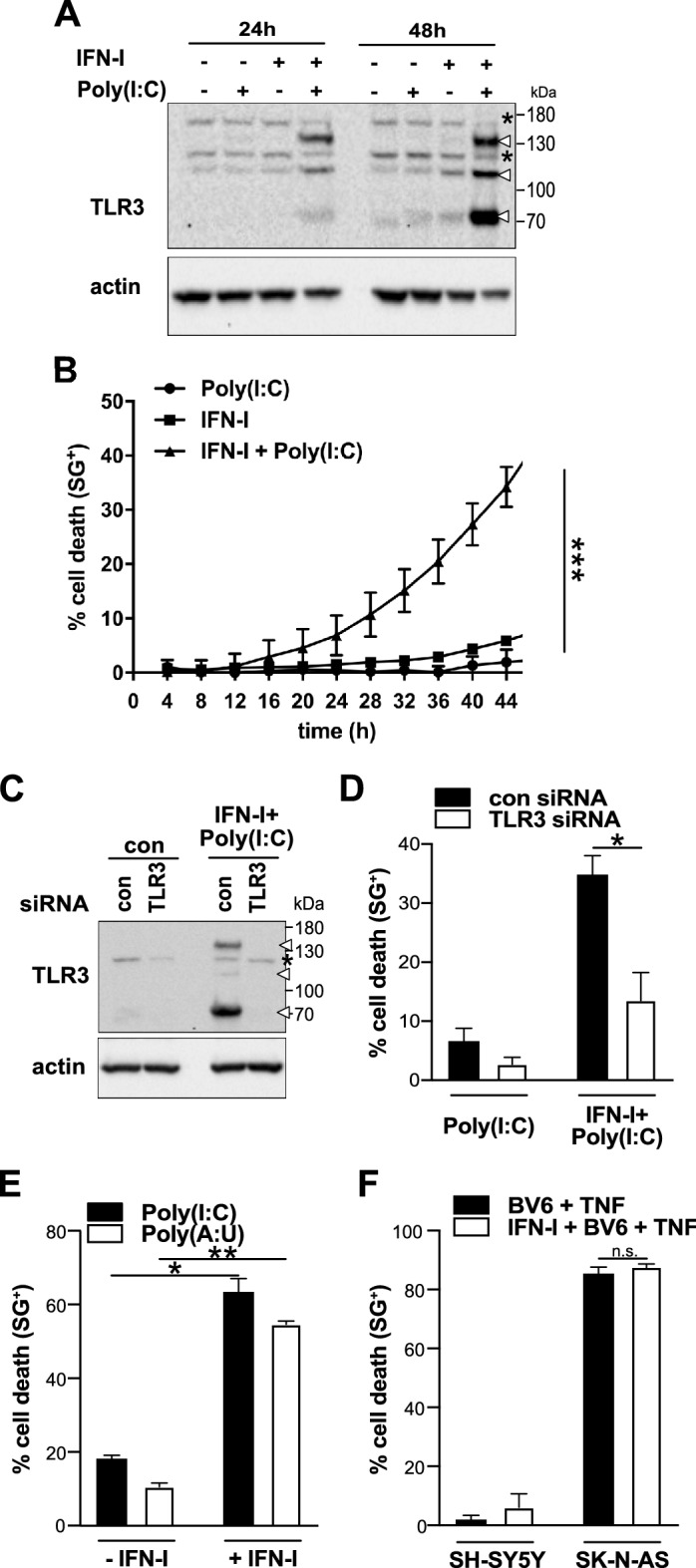
IFN-I/Poly(I:C) treatment induces TLR3-specific cell death in Caspase-8 deficient SH-SY5Y. (**A**) TLR3 expression levels following IFN-I/Poly(I:C) treatment for 24 h and 48 h in SH-SY5Y cells. Asterisks denote unspecific bands. (**B**) Cell death profile of SH-SY5Y cells pre-treated with IFN-I for 16 h prior to Poly(I:C) treatment at the indicated time points. The percentage of SYTOXGreen^+^ (SG^+^) cells was analyzed and profiles are expressed as mean ± S.E.M. n (number of independent experiments) = 3; ***p < 0.001. (**C**) TLR3 expression levels of SH-SY5Y cells following siRNA mediated down-regulation of TLR3 and treatment with IFN-I/Poly(I:C). (**D**) Cell death profile of TLR3 siRNA transfected SH-SY5Y cells following IFN-I/Poly(I:C) treatment for 48 h determined by SYTOXGreen (SG) uptake. Data points represent the mean ± S.E.M of three independent experiment (n = 3); *p < 0.05 (**E**) Cell death profile of SH-SY5Y cells treated with IFN-I/10 μg/mL Poly(I:C) or IFN-I/100 μg/mL Poly(A:U) for 48 h, determined by PI uptake. n = 3; *p < 0.05, **p < 0.01 (**F**) Cell death profile of SH-SY5Y cells treated for 36 h with BV6 + TNF or IFN-I + BV6 + TNF. Cell death was determined by SG positivity. Data points represent 3 independent repeats. ± SEM is indicated. SK-N-AS cells were used as a positive control. *n.s.* non statistical significant.

Activation of TLR3 triggers the extrinsic apoptotic pathway with Caspase-8 as an apical caspase in the Caspase-8 positive, RIPK3 negative Neuroblastoma cell line, SK-N-AS^6^. Since high-grade Neuroblastoma, which are largely therapy resistant, are often characterized by Caspase-8 deficiency, we aimed to investigate whether TLR3 can induce an alternative programmed cell death pathway in the absence of Caspase-8. We studied the Caspase-8 negative NB cell line SH-SY5Y^14^, which originally derived from a metastatic bone tumor biopsy and are a sub-line of the parental line SK-N-SH. Since SH-SY5Y cells are also negative for TLR3, cells were pre-treated with IFN-I for 16 h to restore TLR3 expression followed by treatment with the synthetic ligand of TLR3, Poly(I:C), for indicated times (Fig. 1A).

Alongside the full-length protein, represented by a 130 kD band (*N*-glycosylated form) and a band around 110 kDa, the 70 kDa signaling-competent TLR3 cleavage fragment was readily detectable (Fig. 1A)^15^. In correlation with the detection of TLR3, IFN-I/Poly(I:C) treatment induced a time-dependent increase in the percentage of SYTOXGreen positive cells (SG^+^), which is a nucleic acid stain exclusively permeant to dead cells with comprised membranes (Fig. 1B).

Next to TLR3, Poly(I:C) can also activate, the cytosolic RNA sensors MDA5 and RIG-I^16,17^. In order to verify that observed cell death is a TLR3 specific effect, we silenced the expression of TLR3 by siRNA. Seventy-two hours after siRNA transfection, all three TLR3 forms were absent on Western blot (Fig. 1C), which resulted in reduced cell death induction by IFN-I/Poly(I:C) (Fig. 1D). Results were further strengthened by replacing Poly(I:C) with Poly(A:U), which has a higher affinity for TLR3 than for cytosolic RNA sensors, albeit being less efficient^18^. In SH-SY5Y, treatment with IFN-I/Poly(A:U) induced cell death at a comparable level as treatment with IFN-I/Poly(I:C) (Fig. 1E). TLR3-induced cell death is also not indirectly triggered through an autocrine TNF-α loop since SH-SY5Y cells are not sensitive to TNF-α treatment (Fig. 1F). In line with TNF Receptor (TNFR)-deficiency of SH-SY5Y cells^19^, also pre-treatment with the SMAC mimetic BV6, which is commonly used to switch from TNFR-inflammatory complexes to death complexes did not confer sensitivity to TNF-α (Fig. 1F).

Collectively, these results indicate that IFN-I/Poly(I:C) induces cell death in Caspase-8 and RIPK3 deficient SH-SY5Y cells through specific activation of TLR3 and independently of secondary signaling.

### TLR3-induced cell death does not resemble classical apoptotic or necroptotic cell death

**Figure 2:**
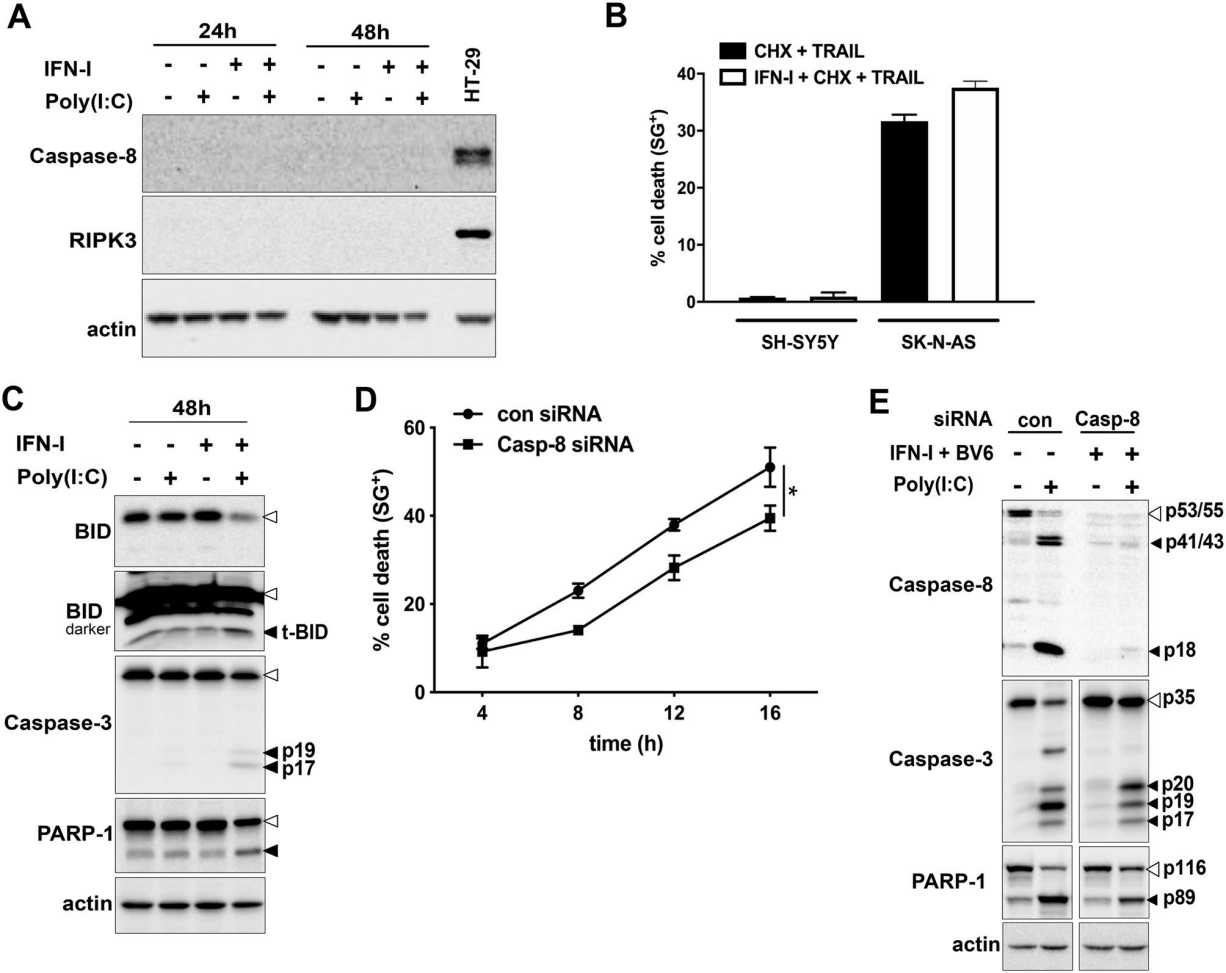
TLR3-induced cell death activates caspases independently of Caspase-8. (**A**) Expression levels of Caspase-8, RIPK3 and MLKL in SH-SY5Y cells following treatment with IFN-I/Poly(I:C) for 48 h. HT29 cells were used as a positive loading control. (**B**) Cell death profile of SH-SY5Y cells treated with IFN-I/TRAIL for 36 h. The percentage of SG^+^ cells was analyzed and profiles are expressed as mean ± S.E.M. n = 2. (**C)** Western blot analyses of the processing of Bid, Caspase-3 and PARP-1 following IFN-I/Poly(I:C) treatment for 48 h. Pro-forms are indicated by an empty arrowhead, while the cleavage products are indicated by black solid arrowheads. Actin was used as a loading control. **(D)** Cell death profile of SK-N-AS cells transfected either with control (con) or Caspase-8 (Casp-8) siRNA for 72 h and treated with BV6 + IFN-I + Poly(I:C). Cell death was determined by SG uptake and data points represent the mean ± S.E.M. of at least three independent repeats. *p < 0.05 **(E)** Caspase-8, Caspase-3 and PARP-1 cleavage following BV6 + IFN-I + Poly(I:C) treatment of SK-N-AS transfected with control (con) or Caspase-8 (Casp-8) siRNA. Pro-forms are indicated by an empty arrowhead, while the cleavage products are indicated by black solid arrowheads. Actin served as loading control.

TLR3 activation induces in tumor cells the extrinsic apoptotic pathway, with Caspase-8 as apical caspase^12,20^. SH-SY5Y cells are Caspase-8 negative^21^ leaving them resistant to extrinsic/receptor mediated apoptosis. However, IFN-γ can up-regulate Caspase-8 expression in SH-SY5Y cells, rendering cells sensitive to TRAIL-induced apoptosis^22,23^. IFN-I did not re-express Caspase-8 in our cellular system (Fig. 2A). Furthermore, IFN-I-treatment also did not sensitize SH-SY5Y cells to TRAIL-induced apoptosis, independently of cycloheximide (CHX) pre-treatment (Fig. 2B)^24^. Despite the absence of the initiator caspase of the extrinsic pathway^21^, cleavage of BID, processing of Caspase-9 and -3 as well as the cleavage of the Caspase-3 substrate PARP-1 were detected following IFN-I/Poly(I:C) treatment (Fig. 2C). Similar results were observed in the NB cell line, SK-N-AS, which endogenously expresses both TLR3 and Caspase-8. In line with an earlier report^6^, activation of TLR3 induced classical apoptosis with Caspase-8 as an apical caspase (Fig. 2D,E). Knock down of Caspase-8 by RNA interference did not abolish TLR3-induced cell death in SK-N-AS (Fig. 2D) with Caspase-3 as well as PARP-1 still being processed (Fig. 2E). This result is in sharp contrast to our earlier finding in lung cancer cell lines, where TLR3-induced cell death is largely inhibited by siRNA-mediated down-regulation of Caspase-8 expression^12^.

Collectively, these results suggest that activation of TLR3 induces a form of cell death which is accompanied with the activation of caspases, albeit Caspase-8 does not appear to be the initiator caspase.

### TLR3-induced cell death is not dependent on caspase activation

**Figure 3:**
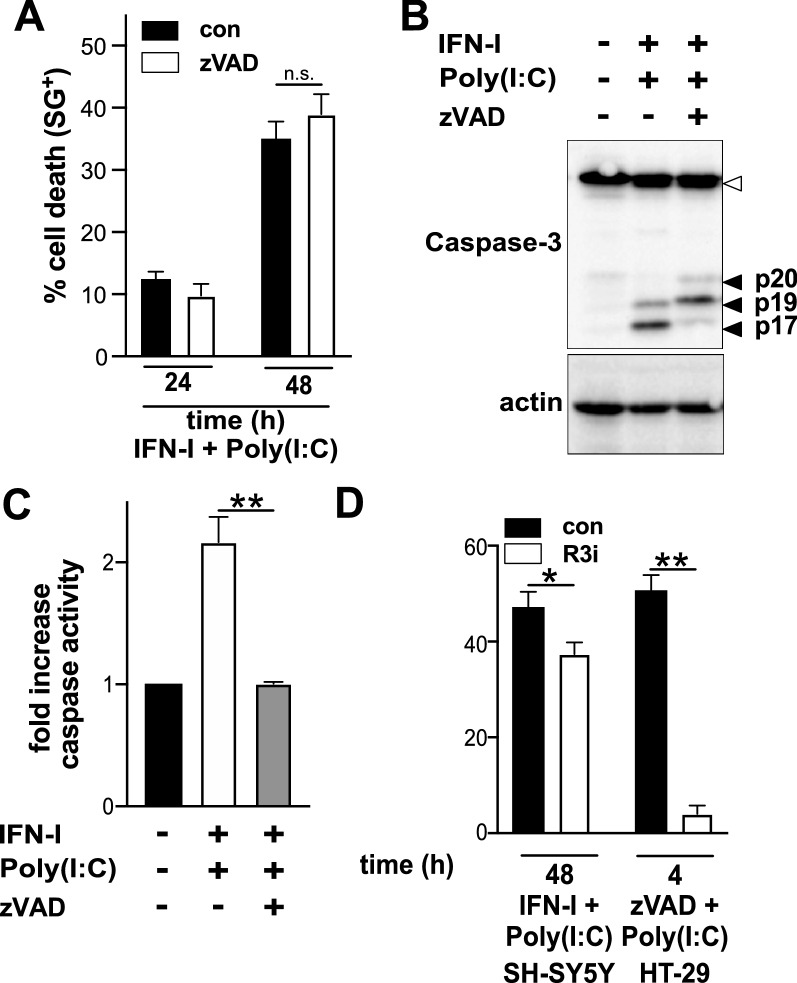
TLR3-induced cell death does not resemble extrinsic apoptosis nor necroptosis. (**A**) Cell death profile of SH-SY5Y cells pretreated with 25 µM zVAD followed by IFN-I/Poly(I:C) treatment. Cell death was determined by SG positivity and data points represent the mean ± S.E.M. of n = 3; non significant (n.s.) (**B**) Caspase-3 cleavage of SH-SY5Y cells pre-treated with 25 µM zVAD followed by treatment for 48 h with IFN-I/Poly(I:C). Caspase-3 pro-forms are indicated by an empty arrowhead, while the cleavage products are indicated by solid black arrowheads. Actin was used as a loading control. (**C)** Caspase-enzymatic activity determined by fluorescence signal emitted due to enzymatic cleavage of Caspase specific substrate DEVD-AFC. The inhibitory effect of 25 µM zVAD concentrations is displayed as fold decrease of the fluorescence signal obtained in SH-SY5Y treated with IFN-1/Poly(I:C) for 48 h. Data points represent the mean ± S.E.M. of at four independent experiments. **p < 0.001 (**D**) Cell death profile of SH-SY5Y cells pre-treated with the RIPK3 inhibitor GSK’872 (R3i) followed by treatment with IFN-I/Poly(I:C). Cell death was determined by SG uptake. Data points represent the mean ± S.E.M. of three independent experiments. HT-29 cells treated with Poly(I:C), BV6 and zVAD were used as positive control of necroptosis induction. *p < 0.05, **p < 0.001.

To determine if caspase activation is required for TLR3-induced cell death execution, the effect of the pan-caspase inhibitor zVAD on TLR3-induced cell death was determined. Interestingly, zVAD had no effect on TLR3-induced cell death in SH-SY5Y cells (Fig. 3A). The formation of the proteolytic active p17 fragment of Caspase-3 as well as cleavage of a fluorescence Caspase-3 substrate were prevented (Fig. 3B,C) demonstrating that caspase activity was efficiently blocked by the zVAD concentrations used. These results support the idea that caspases are not the main executer in TLR3-induced cell death in SH-SY5Y cells.

Thus, it seems that TLR3 induces an alternative caspase-independent programmed death pathway, such as necroptosis. However, necroptosis seems unlikely, as the essential necroptotic key factor RIPK3 is, with or without IFN-I treatment, not expressed in SH-SY5Y (Fig. 2A). In agreement with the absence of RIPK3, the RIPK3 inhibitor GSK’872 (R3i) had no effect on TLR3-induced cell death in SH-SY5Y cells, but efficiently inhibited necroptosis in HT-29 cells (Fig. 3D).

Collectively TLR3-induced cell death in SH-SY5Y cells is distinct from classical extrinsic apopotosis or necroptosis.

### TLR3-induced cell death requirees lysosomal permeabilization

**Figure 4:**
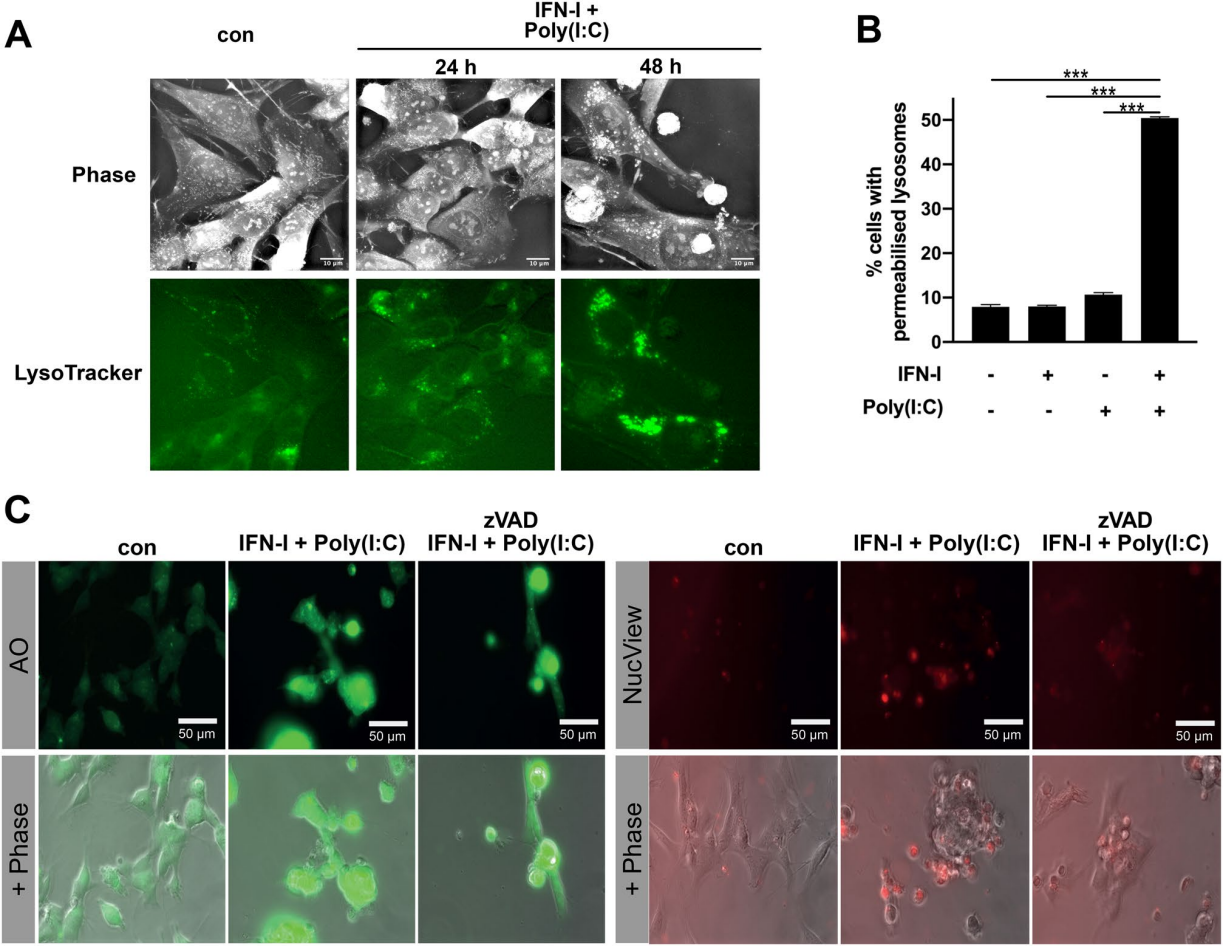
TLR3-induced death involves LMP. (**A**) 2D Refractive index I maps and individual lysosome-specific fluorescence signal (LysoTrackerGreen), after treatment with IFN-I/Poly(I:C) or control for 48 h. Images were generated with Steve Software, Version V1.6.3496, www.nanolive.ch**(B)** LMP evaluated by the percentage of increase in green fluorescence, quantified by flow cytometry, compared with non-treated control using the AO relocation technique. Values represent mean ± S.E.M. *n* = 3; ***p < 0.0015. **(C)** Epifluorescence images of SH-SY5Y cells left untreated or treated with IFN-I/Poly(I:C) or IFN-I/Poly(I:C) + zVAD 25 μM for 48 h and stained with AO (left panels) or NucView (right panel). The bottom panel represents merged images with the corresponding phase.

Based on the intracellular localization of signaling-competent TLR3, another alternative cell death mode to consider is LCD, where permeabilization of the lysosomes releases their deadly content into the cytosol. In line with this idea, treatment with IFN-I/Poly(I:C) increased the number and size of lysosomes (Fig. 4A), which is a prerequisite for their permeabilization^25,26^. Cells with abundant and enlarged lysosomes eventually rounded up and displayed membrane blebbing, which is characteristic for apoptotic body formation. LMP was determined by Acridine Orange (AO) staining, which accumulates in intact lysosomes emitting a red fluorescence which changes to a green fluorescence once it is released in the cytosol. Following IFN-I/Poly(I:C) treatment an increase in green fluorescence intensity was evident, which indicated LMP (Fig. 4B). Together, these results position LMP before the detection of morphological signs of cell death. To further confirm that observed LMP is the cause of cell death, occuring upstream of caspase activation, the relationship between LMP and caspase activation in the presence of zVAD was analyzed (Fig. 4C). IFN-I/Poly(I:C) treatment induced an increase in AO-associated green fluorescence as a readout for LMP. However, this effect was not reverted by zVAD, reinforcing the idea that LMP occurs in a caspase-independent manner. Further, zVAD also did not prevent rounding of the cells, a hallmark of cell undergoing death. The efficient inhibition of caspase-activity by zVAD was determined by absence of NucView red fluorescence, which is a readout for caspase-3 activity. Importantly, also here rounding of the cells was not inhibited by zVAD. In conclusion, LMP appears to be the cause of TLR3-induced cell death, positioning LMP upstream of caspase activation.

## Discussion

Most, if not all, cell death pathways eventually lead to LMP^27^, while we could find both evidence for apoptosis and signs of LMP following TLR3-induced cell death in SH-SY5Y cells. To define if the observed cell death represents LCD, the temporal sequence of apoptotic events and LMP is decisive.

TLR3 is described to act as a death receptor in tumor cells by triggering the extrinsic apoptotic pathway with Caspase-8 as an apical caspase^11,12^. In this scenario, LMP can occur indirectly as a consequence of cell death, while cathepsins released from the lysosomes might amplify cell death signaling^28,29^. In contrast, Caspase-8 can also induce directly LMP in a JNK-dependent manner, which ultimately leads to the activation of the intrinsic apoptotic pathway^30^. However, TLR3-induced cell death in SH-SY5Y cells occurs in the absence of Caspase-8 (and also Caspase-10), excluding any direct or indirect involvement of Caspase-8 in triggering LMP. Another argument for LMP being the cause of cell death is the dispensable role of caspase activity for LMP and the execution of cell death. Consequently, TLR3 turns out to be a master regulator, which can induce different programmed cell death routes, dependent on the availability of key signaling factors. In settings of apoptosis and necroptosis-deficiency, TLR3 activation appears to induce LCD, with LMP being the initiating event.

LMP, if limited, can trigger programmed cell death by mediating the controlled and regulated release of intra-lysosomal proteases, like for example cathepsins. In the cytoplasm, they cleave a wide variety of intracellular targets^24^, such as the BCL-2 protein BID^4,29,31^. At the mitochondria, tBID interacts with other members of the BCL-2 family to induce mitochondria outer membrane permeabilization and subsequent activation of the intrinsic apoptotic caspase cascade^32^. Hence, mechanistically, IFN-I/Poly(I:C)-induced tBID cleavage might provide the molecular link between LMP and the subsequent activation of caspases in SH-SY5Y cells.

In contrast to other death receptors, TLR3 is localized at the lysosomes, and hence, signals where LMP takes place. Thus, TLR3 might directly recruit and interact with factors to mediate regulated LMP in order to release the intra-lysosomal content in a controlled and regulated manner. In this respect, the classical pore forming proteins of the apoptotic pathway, BAK and BAX, and of the necroptotic pathway, MLKL, should be considered as possible pore forming candidates^33,34^. To decipher the precise role of a lysosomal pore in TLR3-induced LCD and its composition will obviously require further investigation.

Programmed cell death pathways are hijacked in cancers, leading to therapy resistance and relapse^35^. The induction of an alternative death pathway to bypass installed resistance mechanisms could guarantee therapeutic success. In this respect, TLR3-induced LMP, which occurs independently of Caspase-8, could represent a novel therapeutic strategy for cancers which silence Caspase-8 expression, like for example neuroblastoma.

## Methods

### Cell culture and treatment procedure

SH-SY5Y as well as SK-N-AS cells (both purchased from ATCC) were cultured in Dulbecco’s modified eagle’s minimal essential medium. HT-29 cells were purchased from ATCC and cultured in McCOY’s medium. Both media were supplemented with 10% fetal calf serum (FCS) and l-glutamine (200 mM). Cell cultures were routinely tested for mycoplasma contamination. SH-SY5Y cells as well as SK-N-AS cells were cultured between a minimal confluency of 40% and a maximal confluency of 70–80%. To trigger TLR3-induced LCD, SH-SY5Y and SK-N-AS cells were pre-treated for 16 h with 1000 Units/mL Type I Interferon (Interferon α/β, R&D Systems, Minneapolis, MN, USA) followed by 2.5 μM BV6 (Selleckchem, Houston, TX, USA) pre-treatment (SK-N-AS cells only) and treatment with 10 μg/mL Poly(I:C) or 100 μg/mL (Poly(A:U)) (both Invivogen). Human TNF-*α* (600 lU/mL) from R&D Systems (Minneapolis, MN, USA) was used at 50 nM and human sTRAIL/Apo2L (PeproTech, Neuilly-Sur-Seine, France) at (200 ng/mL), which a pre-treatment with cycloheximide (CHX, Sigma-Aldrich, Saint-Louis, MO, USA) for 2 h proceeded. The following inhibitors of cell death were used: 25 μM z-Val-Ala-DL-Asp(Ome)-fluoromethylketone (zVAD-fmk, MedChemExpress, New Jersey, USA), 5 μM RIPK3 inhibitor GSK872’ (Selleckchem, Houston, TX, USA).

### Antibodies

The following antibodies (Ab) were used for Western blotting: anti-TLR3 (clones D10F10), anti-Caspase-8 (clone 1C12), and anti-Caspase 3 (# 9662), anti-PARP-1 (# 9542) and anti-BID (# 2002) all from Cell Signaling Technology (Danvers, MA, USA). Anti-actin-HRP was purchased from Abcam.

### RNA interference

Control and human TLR-3 (L-007745-00-0005) and Caspase-8 siRNA ON-TARGET SMARTpool were obtained from Dharmacon. Cells were transfected with the siRNAs using Lipofectamin RNAiMAX (ThermoFisher Scientific, Waltham, MA, USA) according to manufacturer’s instructions for 48 h (Caspase-8) or 72 h (TLR3). The final siRNA concentration was 20 nM for Caspase-8 and 40 nM for TLR3.

### Cell death assay

Cells were stained with 5 μM SYTOXGreen (SG) (Life Technologies) and cell death measured with an Incucyte ZOOM system (Essen BioScience, Michigan, USA). Percent cell death was calculated as follows: [100 × (inducedfluorescence − backgroundfluorescence)] ÷ (maximalfluorescence achieved by Triton-X-100 permeabilization − backgroundfluorescence). The data are presented as mean ± standard error of the mean (S.E.M) of at least 2–3 independent experiments. All cell death profiles in this paper are shown as mean ± S.E.M. For other statistical analysis, two-tailed unpaired *T*-test was used. *P < 0.05; **P < 0.01; ***P < 0.001 were considered as significant.

### Holo-tomographic microscopy (HTM)

SH-SY5Y cells were seeded onto Fluorodishes (ibidi GmbH, Gräfeling, Germany) and stained with 50 nM LysoTrackerGreen (ThermoFisher Scientific, Waltham, MA, USA) 15 min prior to imaging according to manufacturer’s protocol. HTM, in combination with epifluorescence, was performed on the 3D Cell-Explorer Fluo (Nanolive, Ecublens, Switzerland) using a 60 × air objective at a wavelength of λ = 520 nm. Physiological conditions for live cell imaging were reached with a top-stage incubator (Oko-lab, Pozzuoli, Italy). A constant temperature of 37 °C and an air humidity saturation as well as a level of 5% CO_2_ were maintained throughout imaging. Refractory index maps were generated and every 5 min. Images were processed with the software STEVE.

### Lysosomal membrane stability assay

Lysosomal stability was assayed using acridine orange (AO) (Sigma-Aldrich, Saint-Louis, MO, USA) relocation methods. SH-SY5Y cells were loaded with AO (10 μg/mL) for 15 min in complete culture medium at 37 °C prior the end of the 48 h IFN-I/Poly(I:C) treatment. The increase in green fluorescence due to the release of AO from ruptured lysosomes was quantified by flow cytometry in the FL1 channel. Gating strategy is detailed in Supplementary Fig. 1.

### Caspase activity assays

Full cell lysates generated by lysis in RIPA buffer were mixed with caspase reaction buffer (100 mM Hepes pH 7.4, 10% Glycerol, 0.1% CHAPS, 10 mM DTT and 100 μM Ac-DEVD-AFC (from the Caspase-3 Fluorimetric Assay Kit (Biovision K105-400)). Caspase activity (activity/min/mg of protein) was calculated from a 1 h kinetic cycle reading on a spectrofluorometer (380 nm/460 nm or 405 nm/510 nm, Infinite F500, Tecan).

## Supplementary Information

Supplementary Information.
