# Supplementary material for: Caspase-8 deficiency induces a switch from TLR3 induced apoptosis to lysosomal cell death in neuroblastoma
Source: Sci Rep. 2021 May 19;11:10609. doi: 10.1038/s41598-021-89793-1 (PMC8134575; doi:10.1038/s41598-021-89793-1)
Supplement: Supplementary file 1 — Supplementary Information. [file 41598_2021_89793_MOESM1_ESM.pdf]

Caspase-8 deficiency induces a switch from TLR3 induced apoptosis to lysosomal cell death in neuroblastoma cell lines

Marie-Anaïs Locquet[1], Gabriel Ichim[1,2], Joseph Bisaccia[1], Aurelie Dutour[1], Serge Lebecque[1,3], Marie Castets[1]* and Kathrin Weber[1]*

[1]Childhood Cancers and Cell death Laboratory, Cancer Research Center of Lyon (CRCL), INSERM 1052, CNRS 5286, Lyon, France.

[2] Cancer Cell Death Laboratory, part of LabEx DEVweCAN, Cancer Initiation and Tumoral Cell Identity Department, CRCL, Lyon, France

[3]Service D'Anatomie Pathologique, Hospices Civils de Lyon, Hôpital Lyon Sud, Pierre-Bénite, France

*shared last and corresponding authors

Correspondance to: katrin.weber@lyon.unicancer.fr or marie.castets@lyon.unicancer.fr

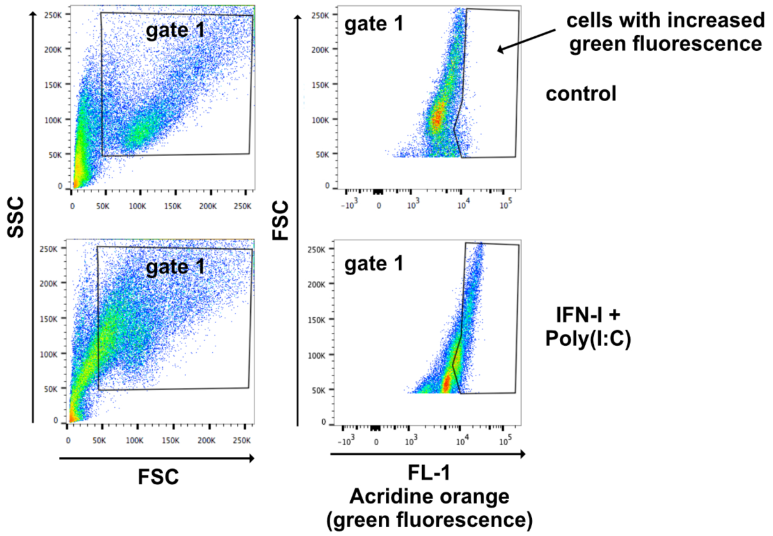

Supplemental Figure 1

Gating strategy to analyze the permeabilization of the lysosome by AO relocalization by flow cytometry. Within the SSC vs FCS density plot “Gate 1” was set to exclude cell debris. Within Gate 1 green fluorescence of AO stained cells was analyzed, whereas the cell population in control condition was set as background green fluorescence intensity and gating performed accordingly. Treatment with IFN-I/Poly(I:C) shifted 47 % into the FL-1 positive gate representing cells with increased green fluorescence compared to control and hence, permeabilized lysosomes.

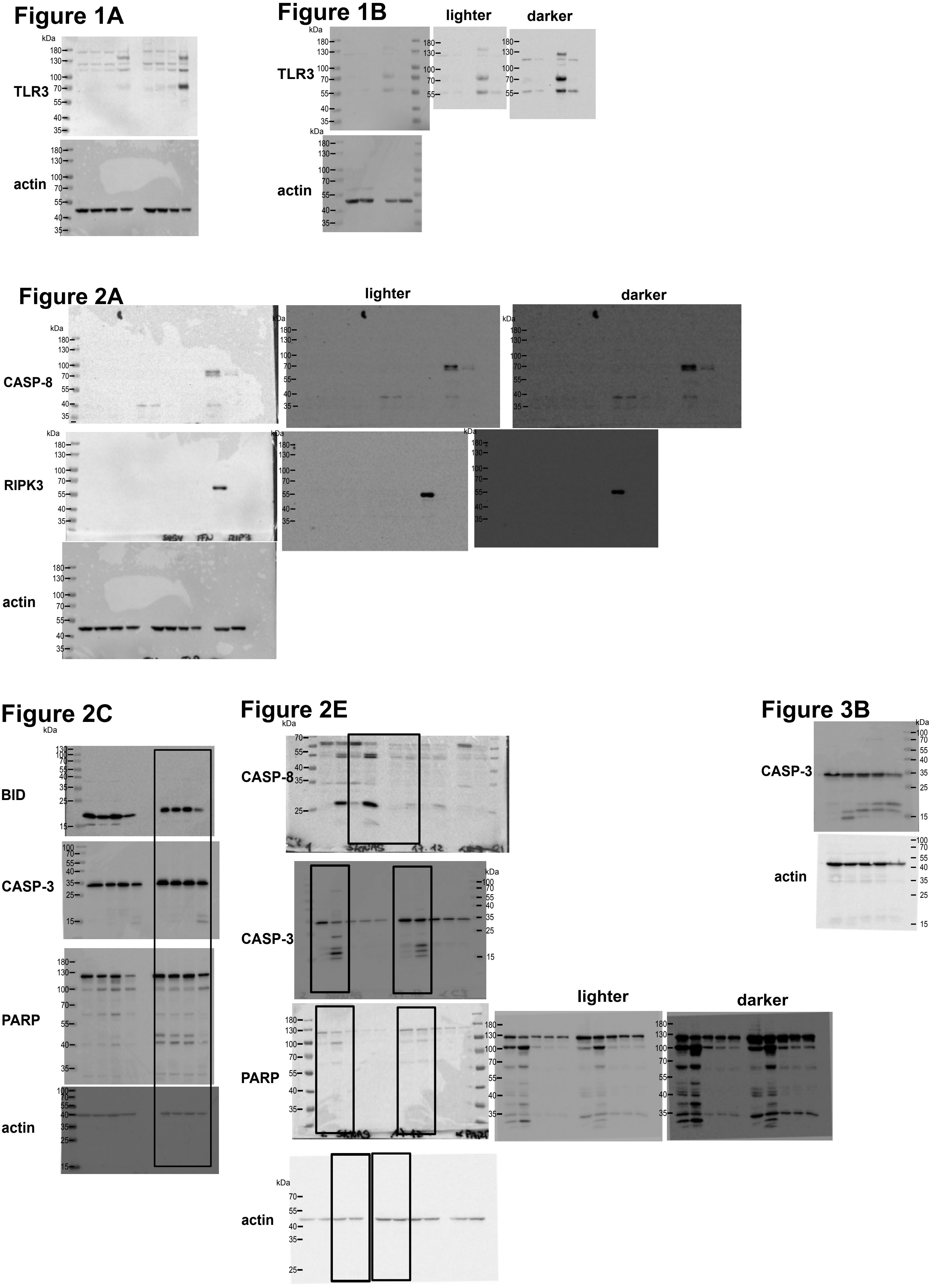

Supplemental Figure 2. Uncroppped Western blots corresponding to Figure 1-3.
